# N-3 Poly-Unsaturated Fatty Acids Shift Estrogen Signaling to Inhibit Human Breast Cancer Cell Growth

**DOI:** 10.1371/journal.pone.0052838

**Published:** 2012-12-28

**Authors:** WenQing Cao, ZhiFan Ma, Mark M. Rasenick, ShuYan Yeh, JiangZhou Yu

**Affiliations:** 1 Department of Pathology and Laboratory Medicine, George Whipple Laboratory for Cancer Research, University of Rochester Medical Center, Rochester, New York, United States of America; 2 Departments of Physiology and Biophysics and Psychiatry, College of Medicine, University of Illinois at Chicago, Chicago, Illinois, United States of America; 3 Jesse Brown VA Medical Center, Chicago, Illinois, United States of America; 4 Department of Urology, George Whipple Laboratory for Cancer Research, University of Rochester Medical Center, Rochester, New York, United States of America; 5 Department of Urology, First Hospital of Shanxi Medical University, Taiyuan, Shanxi, China; Roswell Park Cancer Institute, United States of America

## Abstract

Although evidence has shown the regulating effect of n-3 poly-unsaturated fatty acid (n-3 PUFA) on cell signaling transduction, it remains unknown whether n-3 PUFA treatment modulates estrogen signaling. The current study showed that docosahexaenoic acid (DHA, C22:6), eicosapentaenoic acid (EPA, C20:5) shifted the pro-survival and proliferative effect of estrogen to a pro-apoptotic effect in human breast cancer (BCa) MCF-7 and T47D cells. 17 β-estradiol (E2) enhanced the inhibitory effect of n-3 PUFAs on BCa cell growth. The IC50 of DHA or EPA in MCF-7 cells decreased when combined with E2 (10 nM) treatment (from 173 µM for DHA only to 113 µM for DHA+E2, and from 187 µm for EPA only to 130 µm for EPA+E2). E2 also augmented apoptosis in n-3 PUFA-treated BCa cells. In contrast, in cells treated with stearic acid (SA, C18:0) as well as cells not treated with fatty acid, E2 promoted breast cancer cell growth. Classical (nuclear) estrogen receptors may not be involved in the pro-apoptotic effects of E2 on the n-3 PUFA-treated BCa cells because ERα agonist failed to elicit, and ERα knockdown failed to block E2 pro-apoptotic effects. Subsequent studies reveal that G protein coupled estrogen receptor 1 (GPER1) may mediate the pro-apoptotic effect of estrogen. N-3 PUFA treatment initiated the pro-apoptotic signaling of estrogen by increasing GPER1-cAMP-PKA signaling response, and blunting EGFR, Erk 1/2, and AKT activity. These findings may not only provide the evidence to link n-3 PUFAs biologic effects and the pro-apoptotic signaling of estrogen in breast cancer cells, but also shed new insight into the potential application of n-3 PUFAs in BCa treatment.

## Introduction

Fish oil dietary supplements have become increasingly popular. They are consumed for a variety of ailments as well as for promotion of general health. Population and preclinical studies have suggested that n-3 PUFAs inhibit BCa growth and improve treatment outcomes [1]. Accumulating evidence states that n-3 PUFAs may exert an antitumor action by altering lipid composition of the plasma membrane, which may affect the physical and chemical properties of lipid rafts, consequently, affecting localization of and interactions among signaling components in the microdomains of cell membrane [2]–[4]. Recent studies in breast cancer cells also found that, n-3 PUFA could incorporate different components of the cell membrane to remodel membrane architecture [5], [6]. These suggested a potential mechanism underlying n-3 PUFA anti-cancer effect. N-3 PUFA treatment decreases EGFR signaling [7], and down-regulates CXCR4 signaling in MDA-MB-231 cells [8], which might play the important roles in the anti-BCa effect of n-3 PUFAs. While E2 signaling is crucial for BCa tumorigenesis and progression, fewer studies have addressed how n-3 PUFAs affect E2 signaling and biologic function in BCa cells. It is noteworthy that in the animal studies on chemo-preventive properties of n-3 PUFAs, estrogen does not override the inhibitory effect of high n-3 PUFA diet on BCa growth [9], implying that n-3 PUFAs might abrogate/reduce/reverse the pro-proliferative effect of estrogen.

Estrogen, a mitogen, stimulates cell proliferation and prevents cell death in many different cell types, and is an important risk factor for BCa development [10]. Anti-estrogen therapies have been widely employed to treat hormone dependent BCa. However, laboratory studies have suggested that estrogen stimulates the apoptosis in long-term estrogen deprivation of MCF-7 BCa cells, and switches from being a mitogenic agent to inhibiting growth and inducing apoptosis [11]–[13]. Two potential mechanisms underlying this paradoxical effect of estrogen have been suggested in the studies that can be triggered either through the extrinsic death receptor pathway [12] or via the intrinsic pathway of mitochondrial disruption and release of cytochrome C [11]. Nevertheless, it is not clear how estrogen might promote BCa cell apoptosis.

Based on the above scientific findings, we propose that n-3 PUFAs alter estrogen signaling cascades in BCa cells, and initiate/augment the inhibitory effect of E2 (or compounds binding to membrane E2 receptors) on breast cancer. In this study, we first found that n-3 PUFA treatment initiated the inhibitory effect of E2 on MCF-7 and T47D BCa cell growth, and increased cell apoptosis. While these effects of estrogen were independent of the classical estrogen receptors, ERα or ERβ, they required the presence of the estrogen-sensitive G protein coupled receptor (GPCR), GPER1. Data from this study could lead to novel insights into the usefulness of n-3 PUFAs in the treatment of BCa.

## Materials and Methods

### 1. Materials

Docosahexaenoic acid (DHA, C22:6), eicosapentaenoic acid (EPA, C20:5) and stearic acid (SA, C18:0) (Sigma, St Louis, MO or NU-chek prep, INC. Elysian, MO) were dissolved in ethanol and stored at −80°C for no more than two weeks. 17-β-estradiol (E2), Noble agar, 3-isobutyl-methyanthine, 8-Bromoadenosine-3′,5′-cyclic monophosphorothioate, Rp-isomer (RP-cAMP), Forskolin, and KT5720 were purchased from Sigma (St Louis, MO). 8-CPT-2me-cAMP, G1, selective agonist of GPER1, ICI-182780, and PPT were purchased from TOCRIS bioscience (Ellisville, MI). Antibodies against ERα and GPER1 were from Sigma and GenScript (Piscataway, NJ), respectively. Other reagents were obtained as follows: specific antibodies to GAPDH, phosphorylated EGFR, EGFR pY1068, phosphorylated Erk1/2, phosphorylated AKT (Santa Cruz Biotechnology, Santa Cruz, CA), and Phosph-(Ser/Thr) protein kinase A (PKA) substrate (Cell Signaling Technology, Danvers, MO).

### 2. Cell lines and culture

MCF-7, T47D, and MDA-MB-231 human BCa cell lines were obtained from the American Type Culture Collection (ATCC). BCa cells were cultured in Dulbecco's modified Eagle's medium, supplemented with 10% of FBS and antibiotics in 100 mm cell culture dishes or 6/12–wells cell culture plates. 12 hours before the beginning of all experiments, cells were cultured in Dulbecco's modified Eagle's medium with 5% charcoal treated FBS and antibiotics. In experiments with MTT assay, 10 nM of E2 were used. In other experiments, 5 nM of E2 were employed. Note that there are two ways commonly used to deliver n-3 PUFAs to cells for *in vitro* studies: dissolving n-3 PUFAs in pure ethanol or making n-3 PUFA-BSA mixtures. N-3 PUFAs delivered by either method can inhibit BCa cell growth and interfere with cell signaling, although the concentrations of n-3 PUFAs used are varied [7], [9], [14]–[17]. In this study, n-3 PUFAs were dissolved in pure ethanol. Identical concentrations of ethanol used in experiment treatments were employed for controls.

### 3. Knockdown of GPER1 and ERα receptors in MCF-7 or T47D BCa cells

To transfect the plasmids carrying GPER1 shRNA (Abcam, Cambridge, MA), cells were seeded at 5×10^4^ cells (12-well) or 1 ×10^5^ cells (6-well) concentration in cell culture plate 24 hours prior to transfection. Cells were transfected with lipofectamine 2000 (Invitrogen, Grand Island, NY) according to the instructions from manufacturer. PcDNA3 expression vectors were transfected as control. 12 hours after transfection, the medium was replaced with normal culture medium, and 72 hours later, the indicated experiments were carried out. ERα was knocked down by infecting cells with ERα shRNA lentivirus and this was controlled with a lentivirus containing scrambled shRNA. The knockdown of GPER1 or ERα was verified with Western Blot.

### 4. Cell proliferation assay

In order to study the effect of E2 on n-3 PUFA-treated BCa cell growth, we performed two assays to measure cell proliferation on each of the cell lines mentioned above. All experiments were performed in at least triplicate, as indicated. First, cells were cultured in their usual culture medium for 25 hours, and then replaced with DMEM medium containing 5% charcoal treated FBS.

1. MTT (3-(4,5-dimethylthiazol-2-yl)-2,5-diphenyl tetrazolium bromide) assay [18], [19]. Medium was removed and cells cultured in 12-well plates were washed one time with PBS. 300 µl of serum-free medium and 30 µl of reagent (5 mg/ml) was added, and incubated for one and a half hours in a humidified 5% CO2 incubator at 37°C. The absorbance was measured using a BIORADMicroplate reader at wavelength of 570 and 620 nm. The difference absorbance values at 570 and 620 nm wavelength represented the directly correlation with number of viable cells per well.
2. Anchorage-Independent Growth Assay: Soft agar plates were prepared in six-well plates with a bottom layer of 0.8% Noble agar in serum-free DMEM. The cells were first seeded in 100-mm tissue culture dishes for 24 hours. After trypsinization, 2×10^4^ cells mixed with 0.8% Noble agar in 10% fetal calf serum-supplemented DMEM were seeded as the top agar layer onto the agar plates. Colonies were visualized after six weeks culture by staining with 0.005% crystal violet. Triplicate wells were prepared for each treatment and the experiments were repeated twice.

### 5. Cell apoptosis assay

1. Flow cytometry assay. Cell apoptosis were determined by Annexin V/PI double staining kit (Merck, Calbiochem, San Diego, CA) according to the manufacturer's instructions. Cells were then subjected to analysis by FACSCanto II flow cytometer, and the data were processed with flowjo software (Flowjo, Ashland, OR).
2. Terminal deoxynucleotidyl transferase-mediated deoxyuridine triphosphate nick-end labeling (TUNEL) assay. TUNEL was performed with a kit from Roche Applied Science (Mannheim, Germany) according to the manufacturer's instruction.

### 6. Immunoprecipitation and Western Blot

Immunoprecipitation and Western Blot was done as described previously with modification [20]. Briefly, MCF-7 cells were cultured in 25 ml cell culture flasks for 72 hours with n-3 PUFAs and/or E2, and then washed twice in PBS. The lysate was collected and cleared by centrifuging at 12,000× *g* for 20 min at 4°C. Protein concentration of supernatants was determined by the method of Bradford (Bio-Rad). After adjusting protein concentration to equal amounts for each sample, the supernatant (450 µl) was incubated with agarose beads coated with protein A/G for 1 h at 4°C with continuous gentle inversion. The agarose beads were pulled down by centrifuging at room temperature and discarded. The lysate was then incubated with 5 µl of polyclonal antibody against EGFR for 24 h at 4°C, and then the antibody/lysate mixture was incubated with agarose beads coated with protein A/G for 6 h at 4°C with continuous gentle inversion. After the agarose beads were washed with lysis buffer three times, 50 µl of SDS-PAGE sample buffer was added and the beads were centrifuged again. 20 µl of supernatant was applied onto 10% SDS-PAGE, and the resolved proteins were analyzed on a western blot using a polyclonal antibody against phosphorylated EGFR. Western Blot were performed and quantitated as described previously [20].

### 7. Enzyme-linked immunosorbent assay (ELISA) for cAMP

Cyclic AMP concentration was analyzed using a kit from Cayman Chemical (Ann Arbor, MI) following the manufacturer's instructions.

### 8. Statistics

Data from at least three different independent experiments were analyzed and expressed as mean ± S.E. Significant differences (*p*<0.05) were determined by a one-way analysis of variance or student's t-test using the Prism version 3.0 software package (GraphPad Software Inc., San Diego, CA).

## Results

### E2 inhibits BCa cell growth in n-3 PUFA–treated BCa cells

**Figure 1:**
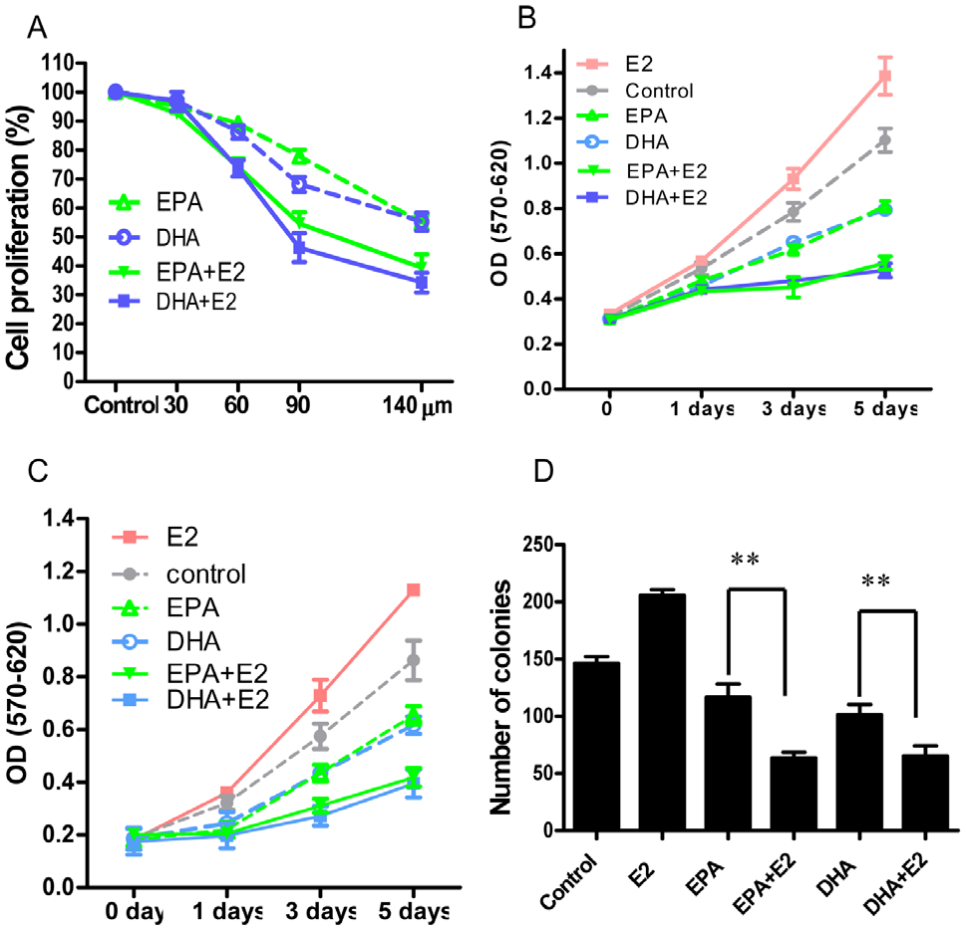
E2 potentiates the inhibiting effect of n-3 PUFAs on BCa cell growth. **A**, Dose response of DHA or EPA in MCF-7 cells with or without E2. The cells were treated with indicated concentrations of n-3 PUFAs for 72 hours ± E2 and cell growth was evaluated by MTT assay (see Material and Methods). Data were normalized to the percent of cell growth in control wells. (n = 5). **B**, Time course for n-3 PUFAs effects in MCF-7 cells. MCF-7 cells were pretreated with 90 µM DHA or EPA with or without 10 nM E2 for different time points (as indicated). Cell growth was measured with the MTT assay (n = 5). **C**, Time course for n-3 PUFAs effects in T47D cells. T47D cells were treated and followed with MTT assay as the same in Figure 1 B. (n = 4). **D**, Quantitated data from Anchorage-Independent Growth Assay (see Methods, n = 3). **p<0.001.

One putative mechanism for n-3 PUFA anti-cancer effects is via alteration of cell-membrane microdomain composition that affects the distribution and function of numerous receptors and other signaling molecules [1], [2], [4]. Thus, we postulated that n-3 PUFA treatment might alter estrogen receptor signaling and its biologic function in BCa cells. In this study, MCF-7 cells were first treated with different concentration of n-3 PUFAs in the presence or absence of E2 for 72 hours (0 to 140 µM). In the line with previous reports, DHA or EPA dose-dependently inhibited MCF-7 cell proliferation (Figure 1 A). The IC50 of DHA or EPA in inhibition of MCF-7 cell proliferation was 173 µM or 187 µM. While E2 treatment did not reduce the inhibitory effect of n-3 PUFAs on BCa cell proliferation, it further decreased the growth of MCF-7 cells (Figure 1 A). The IC50 of DHA or EPA in inhibition of MCF-7 cell growth was decreased when cells treated with E2 (113 µM for DHA+E2, or 130 µm for EPA+E2). The inhibitory effect of E2 on n-3 PUFA-treated MCF-7 cells was time-dependent (Figure 1 B). The saturated fatty acid, stearic acid, did not alter the estrogen pro-proliferative effects in MCF-7 cells (Figure S1 A). The inhibitory effect of E2 on n-3 PUFA-treated BCa cells was also tested with another human BCa cell line, T47D. E2 treatment displayed the same inhibitory effect as that seen in MCF-7 cells (Figure 1 C). The data from MTT assays showed that E2 stimulated MCF-7 cell growth by around 30% in 3-day culture compared to the control group, which is similar with the previous reports using same method to measure cell growth in MCF-7 cells [21]–[23].

Anchorage-independent growth assays were also employed to validate this finding. The number of MCF-7 colonies were significantly decreased in the groups treated only with DHA or EPA compared to the controls. In cells treated with n-3 PUFAs combined with E2, the number of colonies further decreased to 65.3±6.23 or 63.7±4.23 colonies from 101.3±8.1 or 116.7±11.42 colonies per well in cells treated only with DHA or EPA (Figure 1 D). In the absence of n-3 PUFAs, E2 treatment increased cell growth and colony formation (Figure 1 D).

### N-3 PUFA treatment initiates the pro-apoptotic effect of E2 in BCa cells

**Figure 2:**
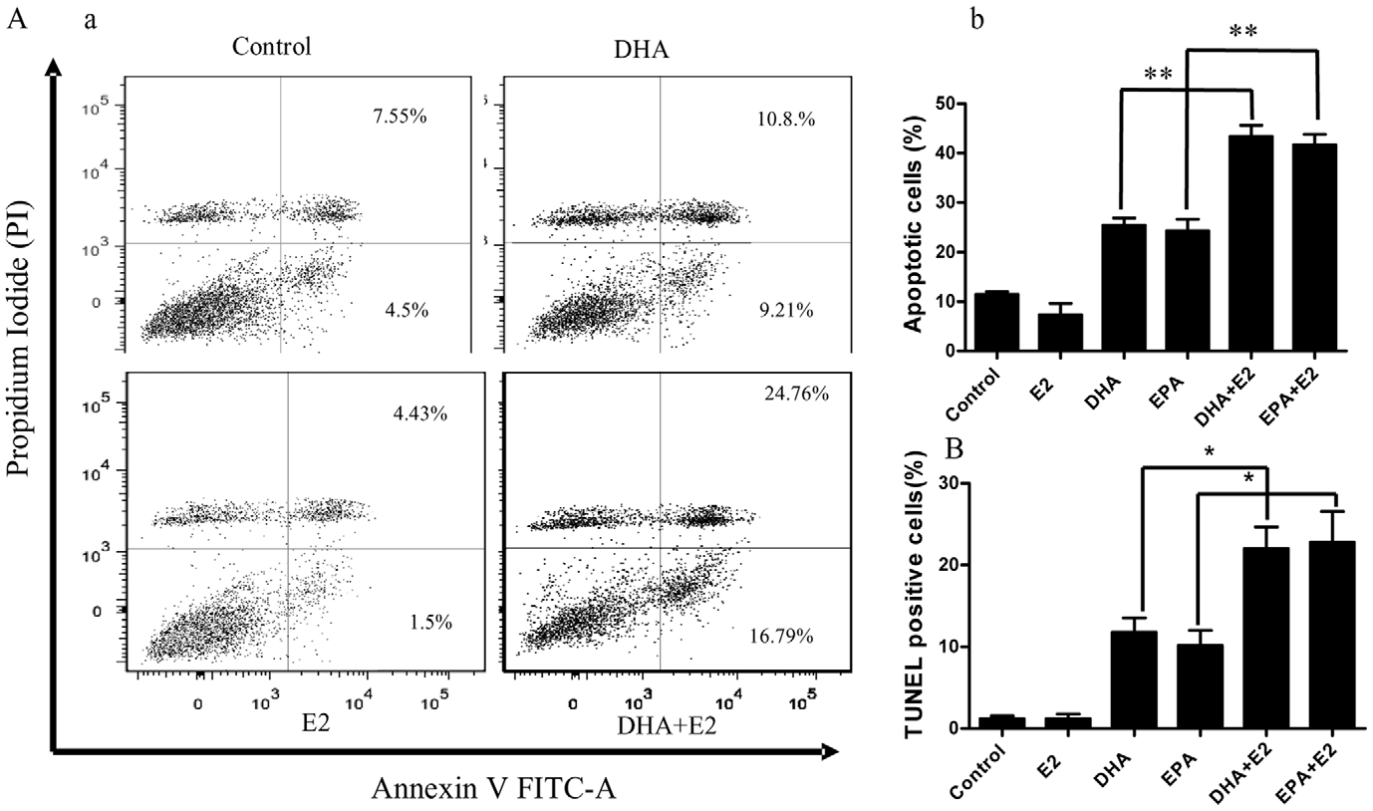
E2 increases the apoptosis of n-3 PUFA-treated BCa cells. **A**. E2 increases DHA-induced apoptosis in MCF7 cells. After 12 -hour n-3 PUFA treatment, E2 was applied, and cells were cultured for 72 hours. Apoptosis was assayed with flow cytometry after staining with PI and Annexin V (see Methods). **a**, Apoptosis was determined after treating MCF-7 cells with E2, DHA (90 µM), or DHA+E2. Flow cytometry profile represents Annexin V staining in x axis and PI in y axis. **b**, Quantitated data include PI^−^/Annexin V^+^ (early indicator of apoptosis) and PI^+^/Annexin V^+^ (later stage apoptosis indicator) cells (n = 3). **B**, TUNEL assay for cell apoptosis. TUNEL-positive cells were stained in nuclei (see s-Figure 1 B). Percentages of TUNEL-positive cells were counted. **C**, E2 decreased Bcl2 expression in MCF-7 cells that were treated with DHA or EPA (90 µM) (n = 3). The mRNA level of Bcl2 was measured with Q-PCR. * *p*<0.05. ** *P*<0.001.

Previous studies have suggested that n-3 PUFAs could inhibit the growth of MCF-7 cells by inducing cell apoptosis [9], [17]. E2 could also promote the apoptosis of BCa cells under certain conditions [24], [25]. Based on the data described above, we proposed that the inhibiting effect of E2 on the growth of n-3 PUFA-treated cells resulted from the E2 pro-apoptotic effect, which might be initiated by DHA or EPA treatment. Flow cytometry assay with Annexin V and PI double staining showed the percentage of apoptotic cells was 21%±2.39 or 20%±2.02 in DHA or EPA treated MCF-7 cell, respectively. The addition of E2 increased the percentage of apoptotic cells to 42%±4.76 or 41.37%±3.21 in DHA or EPA-treated cells (Figure 2 A). TUNEL assays were also employed to measure the apoptosis induced by E2 in the n-3 PUFA-treated MCF-7 cells, which showed similar results as flow cytometry. E2 increased the percentage of TUNEL positive cells in DHA or EPA-treated groups from 11.8%±2.78 or 10.2%±3.29 to 22%±4.28 or 22.8%±4.88 (Figure 2 B, Figure S1 B).

### ERα and ERβ may not play the important roles in the inhibiting effect of E2 on n-3 PUFA-treated BCa growth

**Figure 3:**
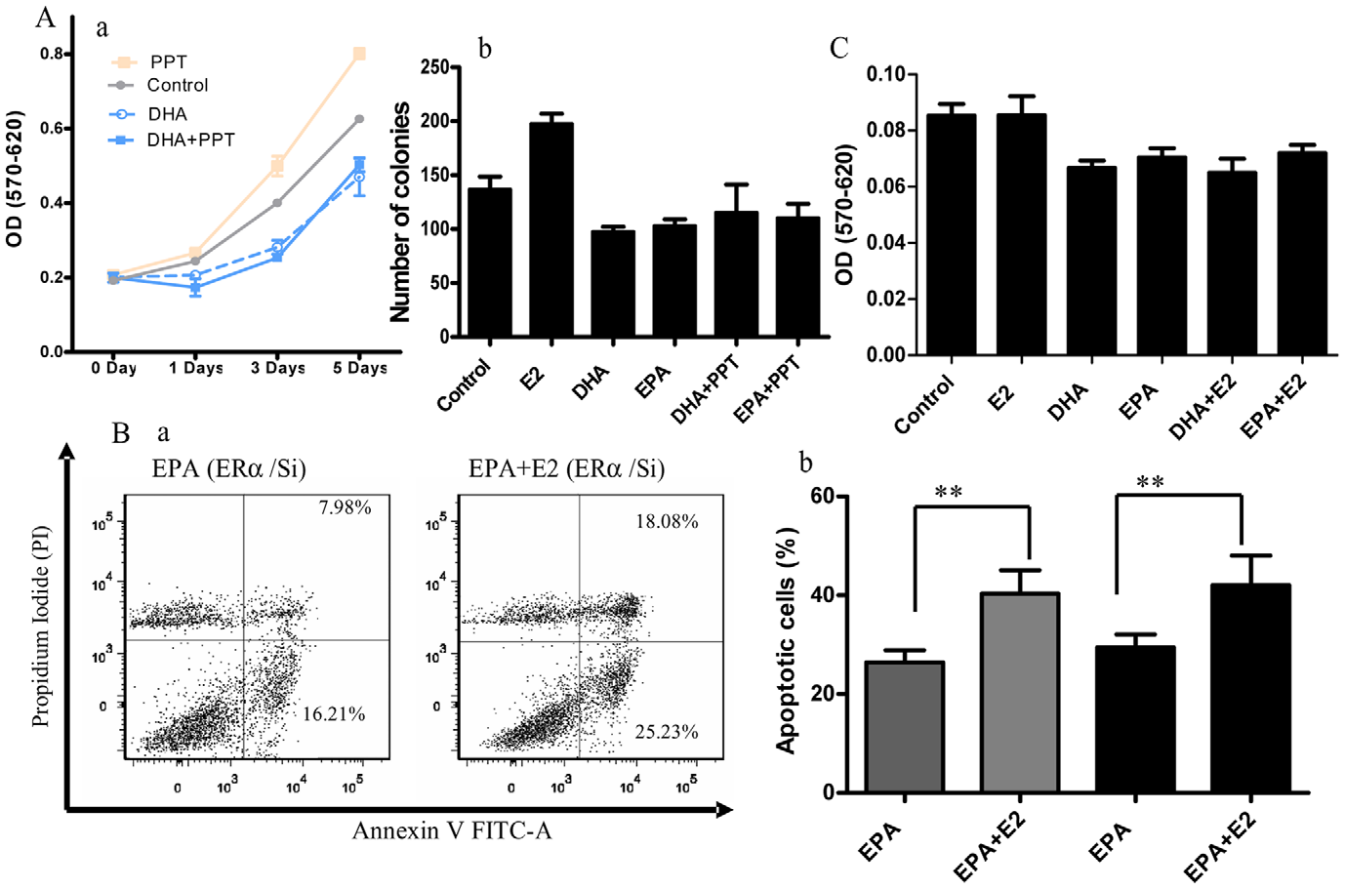
Classical estrogen receptors are not involved in the inhibitory effects of E2 on n-3 PUFA-treated BCa cells. **A**. a, The ERα agonist, PPT (10 nM), did not enhance the inhibitory effect of E2 on T47D (ER^+^) cell growth as measured by MTT assay. b, Anchorage-Independent Growth Assay. **B**, a, Flow cytometry profile represents Annexin V staining in x axis and PI in y axis. The images represented cells expression of ERα shRNA (ERα/si) to silence the ERα expression. b, Quantitated data from flow cytometry assay to show the percentage of apoptotic cells (n = 3). ERα/si and ERα/sc delineated the cells expressing ERα shRNA or its scramble shRNA, respectively. Gray bars indicated the cells expression of ERα scramble shRNA; Black bars delineated the cells expression of ERα shRNA. **C**, Lack of inhibitory effect of E2 on n-3 PUFA-treated MDA-MB-231 cell growth. MDA-MB-231 cells express ERβ, but neither ERα nor GPER1. After 72 hours of treatment with n-3 PUFAs (90 µM) and/or E2 (10 nM), E2 did not affect the n-3 PUFA-treated cell growth (n = 4). **, *p*<0.001, #, *p*>0.05.

Many biologic effects of E2 are mediated by the two known intracellular isoforms of the estrogen receptor (ERα and ERβ) that mainly function as transcription factors for the target genes. A subpopulation of ER localized to the cell membrane or cytoplasm has also been implicated in cell growth and survival [26]. To differentiate E2 receptor types responsible for the observed effects on cell growth, n-3 PUFA- treated T47D cells were incubated with PPT (an ERα selective agonist). PTT stimulated cell growth, but did not affect the cell growth in n-3 PUFA-treated T47D cells (Figure 3 A). This finding suggested that the signals mediated by ERα might not be involved in augmenting n-3 PUFAs inhibition of BCa cell growth. To test this more directly, expression of ERα was knocked down about 85% with shRNA in MCF-7 cells (Figure S2 A). The knockdown of ERα did not significantly reduce the apoptosis induced by E2 treatment in n-3 PUFA-treated MCF-7 cells (Figure 3 B). Moreover, E2 enhancing the inhibitory effect of DHA or EPA (Figure 3 C) was not observed in MDA-MB-231 BCa cells, which express ERβ, but not ERα or GPER1, suggesting that ERβ may also not participate in the inhibitory effect of E2 on BCa cell growth. Notably, GPER1 expression in this cell is inconsistent, even though it is widely reported that MDA-MB-231 cell expresses ERβ, but lacks ERα [27]–[29]. To verify our findings from MDA-MB-231 cells, we tested the expression of ERα and GPER1 with western blot. No protein expression of ERα or GPER1 was detected in the MDA-MB-231 cells used in this study (Figure S2 B).

### GPER1 is involved in the inhibitory effect of E2 on n-3 PUFA-treated BCa cells

**Figure 4:**
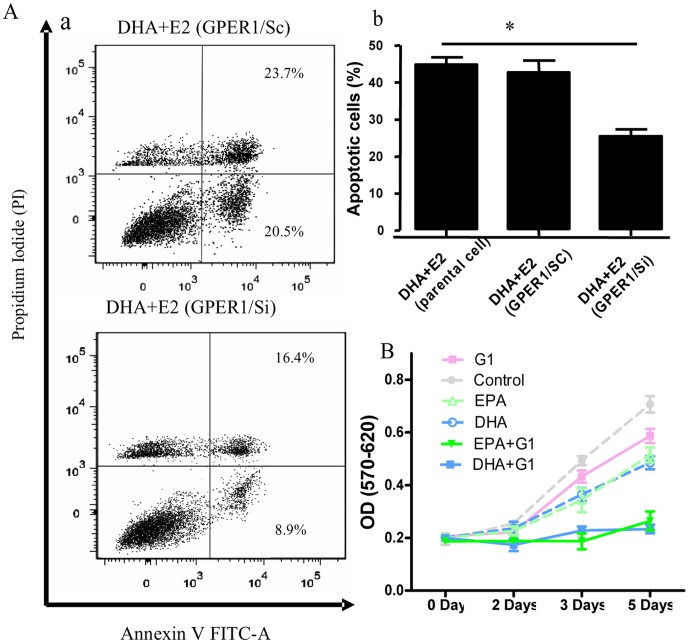
GPER1 may mediate the inhibiting effect of E2 on n-3 PUFA-treated BCa cells. **A**, The GPER1 selective agonist, (G1, 100 nM), mimics the inhibitory effect of E2 on n-3 PUFA-treated BCa cell growth. With n-3 PUFA treatment, G1 significantly suppressed the n-3 PUFA-treated MCF-7 cell growth (n = 4). **B**, GPER1 knockdown in T47D cells. a, Flow cytometry profile represents Annexin V staining in x axis and PI in y axis in T47D. b, Quantitated data from flow cytometry assays showed that GPER1 knockdown inhibited the pro-apoptotic effect of E2 (n = 3). GPER1/si and GPER1/sc indicated cells were respectively transfected with plasmid that carries GPER1 shRNA or its control plasmid. *, *P*<0.05.

The signaling pathways mediating E2 stimulation are mainly consisted with classical signaling pathways through ERα/ERβ and nonclassical signaling pathway via activation of GPER1 or membrane associated ERα/ERβ [30]–[33]. The above experiments suggested that classical ERs might not play an essential role in mediating the E2 anti-cancer effect in n-3 PUFA-treated BCa cells. However, GPER1 was reported to bind E2 with high affinity [30], [31], to mediate the non-classical signaling of E2, to influence growth factor signaling pathways including transactivation of the EGFR, PI-3 kinase translocation, Src activation, Erk activation, cAMP signaling [32], [33], and to modulate downstream transcription factor networks [34]. We postulated that GPER1 might play the important roles in inhibitory effect of E2 on the n-3 PUFA-treated breast cancer cell growth. Concordant with the previous reports [35], G1, a selective agonist of GPER1, suppressed MCF-7 cell proliferation in a time dependent manner. Furthermore, in n-3 PUFA pre-treated MCF-7 cells, G1 further inhibited cell growth (Figure 4 A). The findings suggested that GPER1 might be mediating the additional inhibitory effect of E2 in n-3 PUFA-treated cells. In another BCa cell line, T47D, GPER1 was knocked down (about 70%, Figure S2 C) with GPER1 shRNA. Apoptosis induced by E2 was evaluated in DHA-treated cells with flow cytometry. The results showed that the percentage of apoptotic cells induced by E2 was decreased from 42%±4.98 in cells expressing of control vector to 26%±2.45 in cells that GPER1 was silenced by GPER1 shRNA (Figure 4 A), suggesting that GPER1 plays an important role in the inhibitory effect of E2 on n-3 PUFA-treated BCa cells. Taken together, these results suggest that GPER1 mediates the proapoptotic effect of E2 in n-3 PUFA-treated BCa cells.

### N-3 PUFA treatment blunts the activation of EGFR, Erk1/2, AKT signaling by E2 stimulation

**Figure 5:**
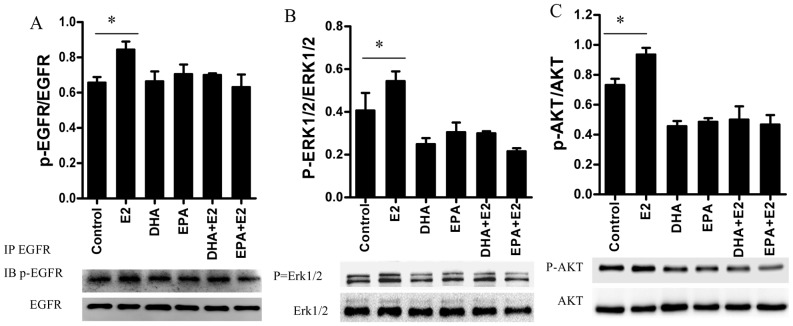
N-3 PUFA treatment blocks the activation of EGFR, ERK and AKT by E2 in MCF-7 cells. Cells were pretreated with n-3 PUFAs for 24 hours, and then replaced the medium containing 5 nm E2 and n-3 PUFAs for 30 min. Cell lysate were collected and processed for immunoprecipitation and Western Blot. **A**, EGFR was immunoprecipitated with EGFR antibody. The phosphorylated EGFR was detected using specific antibody with Western Blot. The upper panel is the quantitated data. The level of phosphorylation of EGFR (p-EGFR) was represented by the ratio of the optical density of p-EGFR and total EGFR. The lower panel is a representative western blot from one of the three similar experiments. **B.** phosphorylated Erk1/2 (p-ERK1/2) was detected with Western Blot. The upper panel is the quantitated data from three individual experiments. The level of phosphorylation of EGFR was represented by the ratio of the gray value of p-Erk1/2 and total Erk1/2. The lower panel depicts one of the three western blots. **C.** Phosphorylated AKT was measured with Western Blot. The upper panel shows the quantification of data from three individual experiments. The level of phosphorylation of AKT was represented by the ratio of the gray value of p-AKT and total AKT. The lower panel is one of the three western blots from three experiments. p-AKT delineated the phosphorylated AKT. *, *p*<0.05.

It is now widely appreciated that estrogens can function through a variety of signaling pathways, such as mitogen-activated protein kinases (MAPK), phosphotidylinositol 3-kinase (PI3K), PKA, EGFR, and IGF. These non-classical manifestations of E2 signaling could be cell-type specific. In BCa cells, EGFR, Erk1/2 and AKT seem to be activated by both membrane-bound ER and GPER1 [26], [36]. Consistent with the previous reports [37]–[39], E2 increased phosphorylated EGFR, Erk1/2 and AKT in this study (Figure 5), which might participate in the pro-proliferative effect of E2. However, n-3 PUFAs decreased the extent of phosphorylated Erk1/2, and phosphorylated AKT, but did not affect phosphorylated EGFR compared to vehicle treatment in MCF-7 cells, suggesting potential molecular targets for n-3 PUFA inhibition of BCa cell growth. Subsequently, phosphorylated EGFR, Erk1/2, and AKT were examined in cells treated with n-3 PUFAs combined with E2. E2 treatment neither stimulated the phosphorylation of EGFR, Erk1/2, and AKT, nor further decreased this property (Figure 5) compared to n-3 PUFA treatment alone.

### GPER1-cAMP-PKA signaling activity is increased by E2 in n-3 PUFA-treated BCa cells, and contributes to the inhibitory effect of E2 on BCa cell growth

**Figure 6:**
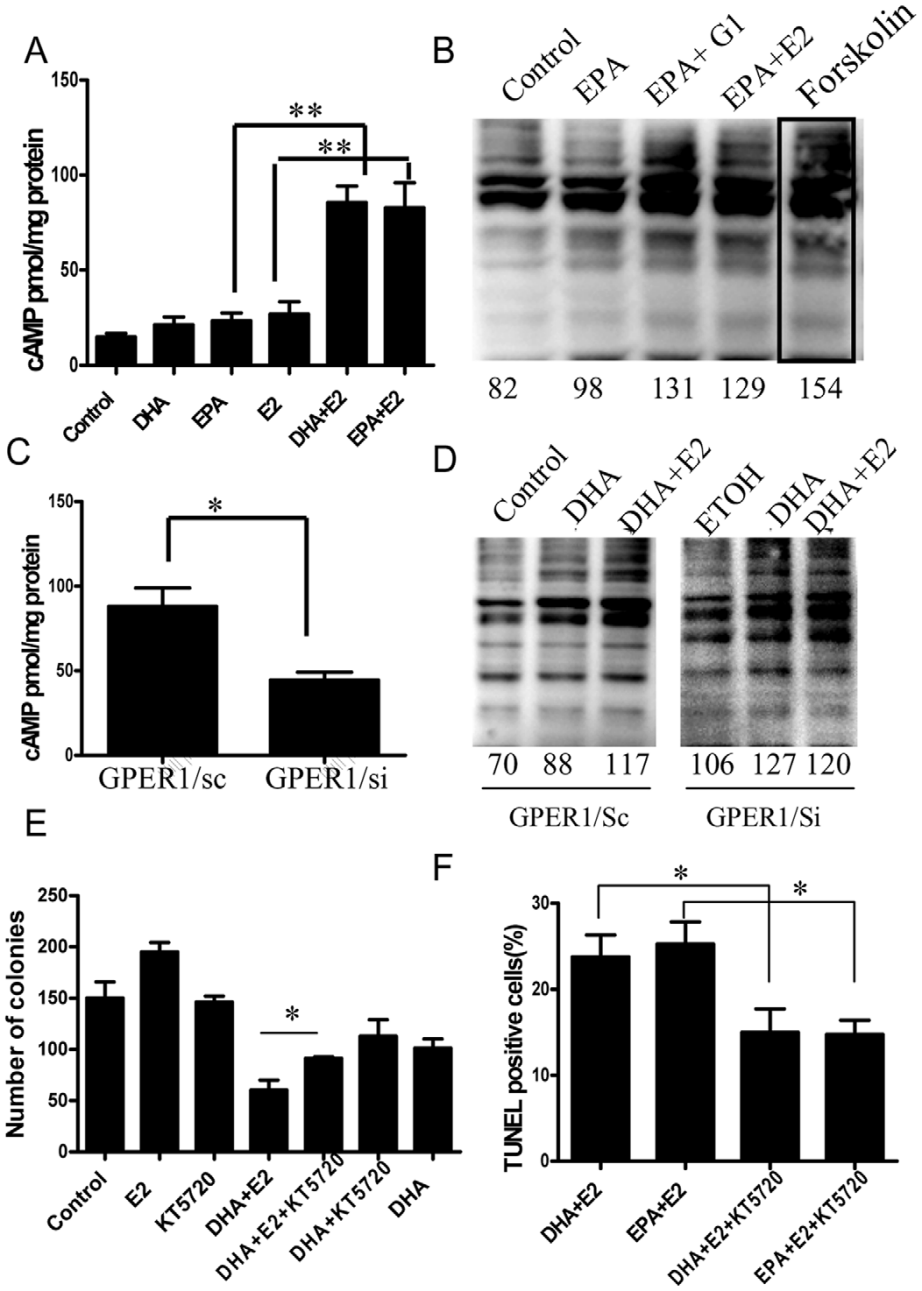
Synergy between GPER1 and n-3 PUFAs in increasing MCF-7 cell cAMP-PKA signaling. **A**. E2 increased intracellular cAMP in n-3 PUFA-treated MCF-7 cells. Cells were treated with DHA or EPA (90 µM) for 24 hours, and then 5 nM E2 for 30 minutes. Cells were then processed for cAMP measurement (see Material and Methods, n = 4). **B**, E2 treatment increased PKA activity. T47D cells were treated in 6-well culture plates as in A, followed by western blot with phosph-(Ser/Thr) substrate antibody. The mean of gray value from the phosphorylated substrate bands in selected rectangle area was measured (see bottom of blot), and represented the PKA activity. One sample of rectangle area was showed in forskolin treated line. The blot represents one of the two separate experiments. Forskolin treatment is a positive control. **C**, Knockdown of GPER1 with GPER1/si reduced cAMP production stimulated by DHA+E2 (left) (n = 3) and **D**, Knockdown of GPER1 with specific shRNA reduced the PKA activity induced by E2 in DHA-treated cells. Expression of vector control (GPER1/sc) or GPER1 shRNA (GPER1/si). The mean of gray value was measured as described in B (see bottom of blot), which indicated the PKA activity (n = 2). **E**, a PKA inhibitor, KT5720 (100 nM), reduced the inhibitory effect of E2 on n-3 PUFA-treated T47D cell colony formation (n = 2). **F**, TUNEL assays showed that in DHA-treated MCF-7 cells, KT5720 decreased the apoptosis induced by E2 (n = 3). *, *p*<0.05.

The published studies have indicated that GPER1 coupled to Gαs and activated cAMP signaling pathways [32], [33]. The results in figure 4 have showed GPER1 mediated the inhibitory effect of E2 on the n-3 PUFA-treated BCa cell growth. In addition, the previous study demonstrated that activation of Gαs-cAMP-PKA signaling inhibited BCa growth *in vitro* and *in vivo* [40]. Thus, it was proposed that the effect of E2 on n-3 PUFA-treated BCa cells was mediated, primarily, by the GPER1-Gαs-cAMP-PKA signaling pathway. To test this, we first measured the production of cAMP. Results showed that n-3 PUFAs or E2 treatment alone, the cAMP had a slight increase compare to the vehicle group. However, E2 significantly increased cAMP production in n-3 PUFA-treated MCF-7 cells (Figure 6 A). Subsequently, antibody against phospho-(Ser/Thr) PKA substrate was used to detect the PKA activity. Consistent with cAMP findings, EPA alone increased the phosphorylated (Ser/Thr) PKA substrates compared to the vehicle treatment. Notably, E2 treatment further increased PKA activity in EPA-treated T47D cells (Figure 6 B). These results indicated that E2 greatly increased cAMP-PKA signaling activity in BCa cells treated with n-3 PUFAs. To test if the alteration of cAMP-PKA signaling induced by E2 in n-3 PUFAs treated BCa cells was mediated by GPER1, GPER1 expression was knocked down in MCF-7 cells using specific GPER1 shRNA as above described in T47D cells. The cAMP production promoted by DHA+E2 in n-3 PUFA-treated MCF-7 cells was attenuated following knockdown of GPER1 (Figure 6 C). Moreover, PKA activity stimulated by E2 in DHA-treated MCF-7 cells was also suppressed by GPER1 knockdown (Figure 6 D).

The role of cAMP-PKA signaling in inhibiting effect of E2 on the n-3 PUFA-treated BCa growth was further evaluated with selective PKA inhibitors. KT5720 (100 ng/ml), a cell-permeable PKA inhibitor, reduced the inhibitory effect of E2 on n-3 PUFA-treated T47D cells. The number of colonies in the DHA+E2+KT5720 treatment group increased to 90.3±7.31 colonies per well from 60.1±6.59 colonies per well in groups treated with DHA+E2 alone. KT5720 itself did not significantly affect the growth of T47D cells (Figure 6 E). To confirm a PKA role in the inhibitory effect of E2 on n-3 PUFAs-treated MCF-7 cell growth, we employed another competitive inhibitor of cyclic AMP-dependent PKA, RP-cAMP. RP-cAMP showed a similar effect as KT5720 to reduce the inhibitory effect of E2 on n-3 PUFAs-treated MCF-7 cells (Figure S3 A). KT5720 also reduced the percentage of TUNEL positive cells promoted by E2 in n-3 PUFAs-treated MCF-7 cells (Figure 6 F). These data strongly indicated that GPER1-cAMP-PKA signaling played an important role in inhibitory effect of E2 on BCa cell growth. In addition, the selective agonist of EPAC, 8-CPT-2me-cAMP, did not mimic the inhibitory effect of E2 on the n-3 PUFA-treated MCF-7 (Figure S3 B), suggesting that the cAMP/EPAC pathway is not involved in the pro-apoptotic effect of E2 on n-3 PUFA-treated BCa cells. n-3 PUFA treatment did not alter the protein expression of ERα and GPER1 in MCF-7 cells (Figure S3 C).

## Discussion

This study demonstrates how n-3 PUFA treatment shifts the pro-proliferative effects of E2 to pro-apoptotic effects in BCa cells. While n-3 PUFAs themselves exerted cytotoxic effects, there was clear synergy with E2. These actions of E2 appear to be via the activation of GPER1 and the subsequent engagement of the cAMP-PKA signaling pathway. It is important to note that Tamoxifen, a major estrogen selective modulator for breast cancer treatment, has been identified as an agonist of GPER1 [27]. Since n-3 PUFAs enhance/promote GPER1-cAMP-PKA signaling pathway response to E2, which mediate the inhibitory effect of E2 on ER^+^ BCa cell, it is possible to employ n-3 PUFAs to strengthen the anti-cancer effect of Tamoxifen. In addition, There is a high risk of breast cancer for the post-menopause women who take hormone replace therapy [41]. N-3 PUFAs may provide prevention for the vulnerable population through shift the pro-proliferative effect of estrogen to its pro-apoptotic effect.

Estrogen inhibition of BCa growth and induction of apoptosis have been reported previously [24], [42]–[44]. Song and other investigators demonstrated that E2 induced apoptosis in hormone-dependent BCa cells that underwent long-term estrogen deprivation [12], [44]. Moreover, the studies also revealed that in certain anti-hormone resistant BCa cells, E2 treatment triggered the occurrence of apoptosis *in vitro* and *in vivo*, even at physiologic concentrations [11], [45]. Our results showed that n-3 PUFA treatment might represent another specific condition that can initiate the inhibitory effect of E2 on BCa cells.

The mechanism by which estrogen promotes BCa cell apoptosis is not understood. Previous studies from MCF-7 cells with long-term estrogen deprivation or anti-estrogen resistant cells have connected E2-induced apoptosis with activation of the FasR/FasL death-signaling pathway and mitochondrial pathway, suggesting ERα might participate in the pro-apoptotic effect of E2 [11]. However, our data from selective ERα agonists or knockdown of ERα seemed to suggest that ERα did not play the major roles in mediating the pro-apoptotic effect of E2 on n-3 PUFA-treated BCa cells. In addition, E2 did not inhibit MDA-MB-231 cell growth that expresses ERβ, but not has ERα and GPER1, suggesting ERβ might be not involved, either. These studies also raised the possibility that different signaling pathways mediate E2-induced apoptosis in BCa cells under different circumstances. It will be interesting to determine whether n-3 PUFA treatment negatively impacts the transcriptional activity of ERα/ERβ in the future.

Since our data appeared to preclude a role of ERα/ERβ in the effects of E2 on the n-3 PUFA-treated BCa cells, nonclassical steroid actions initiated at the cell surface were studied., In BCa cells, these include many cellular activities, foremost among which are: **1**) Activation of Erk and AKT signaling, transactivation of EGFR, leading to activation of MAPK/Erk cascades and **2**) Stimulation of secondary messengers such as cAMP and calcium signaling pathways through activation of G protein signaling [32]. Increased phosphorylated EGFR, Erk1/2 or AKT may contribute to the pro-proliferation and pro-survival effects of E2 [2], [7]. Our data support this concept because E2 treatment, in the absence of n-3 PUFAs, promoted the phosphorylation of EGFR, Erk1/2 and AKT in MCF-7 cells. Consistent with a previous study in MDA-MB-231 cells [7], n-3 PUFA treatment decreased the phosphorylation of Erk1/2 and AKT. However, unlike MDA-MB-231 cells, n-3 PUFA treatment did not obviously affect the phosphorylation of EGFR in MCF-7 cells. The difference might result from the genetic differences, such as the lack of ER and GPER1 in MDA-MB-231 cells. Furthermore, activation of EGFR, Erk1/2 and AKT stimulated by E2 were blunted by n-3 PUFA treatment; E2 did not significantly affect phosphorylation of EGFR, Erk1/2, and AKT in n-3 PUFA-treated MCF-7 cells. The data suggest that blunting E2 the effect of E2 on EGFR, Erk1/2, and AKT signaling may be one of the mechanisms underlying the pro-apoptotic effect of E2 in n-3 PUFA-treated BCa cells.

Increasing evidence suggest that activation of GPER1 is able to trigger nonclassical estrogen action [30], [46], [47], even though there still are inconsistencies surrounding this [31], [48]. Activation of GPER1 with the selective agonist, G1, seems to affect BCa cell growth in a cell specific manner, because G1 stimulated cell proliferation in ER^−^/GPER1^+^ SKRB3 BCa cells [34], but inhibited the ER^+^/GPER1^+^ MCF-7 cell growth [35]. This suggests that different signaling pathways mediate GPER1 signaling in ER^−^/GPER1^+^ and ER^+^/GPER1^+^ BCa cells. In this study, both MCF-7 and T47D cells are ER+/GPER1+, G1 treatment further inhibited growth of the n-3 PUFA-treated MCF-7 cells, suggesting GPER1 might mediate the pro-apoptotic effect of E2 in the cells. Knockdown of GPER1 attenuated the apoptotic effect of E2 in n-3 PUFA-treated T47D BCa cells; these data are consistent with GPER1 mediating the pro-apoptotic effect of E2.

GPER1 was first identified to bind to Gsα and activate cAMP signaling [27], [49]. cAMP inhibition of BCa growth and promotion of BCa cell apoptosis are well established. Constitutively activated Gsα, when overexpressed in estrogen-dependent human BCa cells, inhibits the ability of these cancer cells to form tumors in athymic mice [50]. Elevation of cAMP can also inhibit BCa cell growth and induce cell apoptosis [51]–[54]. Data from this study showed that E2 significantly increased cAMP production and PKA activity in n-3 PUFA-treated BCa cells (Figure 6), and knockdown of GPER1 abolished the increase of cAMP production and PKA activity induced by E2. Furthermore, the selective PAK inhibitors revised the effect of E2 on cell growth and apoptosis in n-3 PUFA-treated BCa cells. These suggested that the inhibitory effect of E2 on n-3 PUFA-treated BCa cells was mediated by GPER1, through activation of cAMP-PKA signaling. A recent study suggested that the expression of the pro-apoptotic protein BIM might mediate the cAMP/protein kinase A (PKA)-induced apoptosis of immature T cells [55], and this is a possibility for BCa cells as well.

Many studies showed that manipulating cell microdomains like lipid rafts with genetic or chemical techniques modified G protein signaling, and raft disruption either promoted or attenuated signaling, depending upon agonist and G protein [56] [57]–[59]. Lipid composition of the plasma membrane is changed subsequent to incorporation of n-3 PUFAs (a process requiring chronic treatment similar to that employed in this study), which may affect the physical and chemical properties of lipid rafts, subsequently altering distribution and coupling of signaling molecules [2]–[4] [6], [14]. We hypothesize that modulation of non-classical signaling of E2 by n-3 PUFA treatment in BCa cells results from such altered distribution of signaling components such as Gαs, EGFR, and Src kinase between lipid raft and non-raft microdomains. Although beyond the scope of this study, future studies might identify the precise link between n-3 PUFA treatment, lipid rafts and GPER1-cAMP-PKA signaling in BCa cells.

In summary, this study shows, for the first time, that n-3 PUFA treatment shifts the pro-proliferation and pro-survival effect of E2 to a pro-apoptotic effect on BCa cells. The alteration of E2 non-classic signaling resulting from n-3 PUFA treatment may play an important role in mediating the inhibitory effect of E2, in which n-3 PUFA treatment blunts the effect of E2 on EGFR, Erk1/2, and AKT, while increasing GPER1-cAMP-PKA signaling. These findings may shed new insight on the potential treatment of BCa using n-3 PUFAs, and give rise to the possibility to treat BCa through initiating the pro-apoptotic effect of estrogen signaling.

## Supporting Information

Figure S1 **A,** Stearic acid (SA) does not promote the inhibitory effect of E2 in breast cancer cells. SA (90 µM) treated for indicated time points with or without E2. **B,** Images of MCF-7 cells after TUNEL Assay. MCF-7 cells treated as indicated for 72 hours, TUNEL assay was performed as described in Methods. Arrows indicated TUNEL positive cells.
(PPT)

Figure S2 **Protein expression in breast cancer cell.** **A**, Western blot showed the expression of ERα in T47D cells after infected with lentivirus to deliver ERα or scramble shRNA. **B**, Western blot showed the expressions of ERα and GPER1 in indicated human breast cancer cell line. **C**, Western blots showed knockdown of GPER1 after transfection of GPER1 shRNA or control.
(PPT)

Figure S3 **MTT assay for BCa cell growth.** **A**, MTT assay showed that 8-Bromoadenosine-3′,5′-cyclic monophosphorothioate, Rp-isomer (RP-cAMP, 10 uM), another PKA inhibitor, reversed the inhibitory effect of E2 on n-3 PUFA-treated MCF-7 cells (n = 3). **B**, 8-CPT-2me-cAMP (CT-cAMP), an agonist of cAMP-Epac signaling did not mimic the inhibitory effect of E2 on the n-3 PUFA-treated BCa cells (n = 3). **C**, n-3 PUFAs did not alter the ERα and GPER1 expression in n-3 PUFAs treated MCF-7 cells. MCF-7 cells were treated with DHA or EPA with/without E2 for 3 days.
(PPT)
