# Supplementary figures and images for: N-3 Poly-Unsaturated Fatty Acids Shift Estrogen Signaling to Inhibit Human Breast Cancer Cell Growth
Source: PLoS One. 2012 Dec 28;7(12):e52838. doi: 10.1371/journal.pone.0052838 (PMC3532062; doi:10.1371/journal.pone.0052838)

## Slide 1
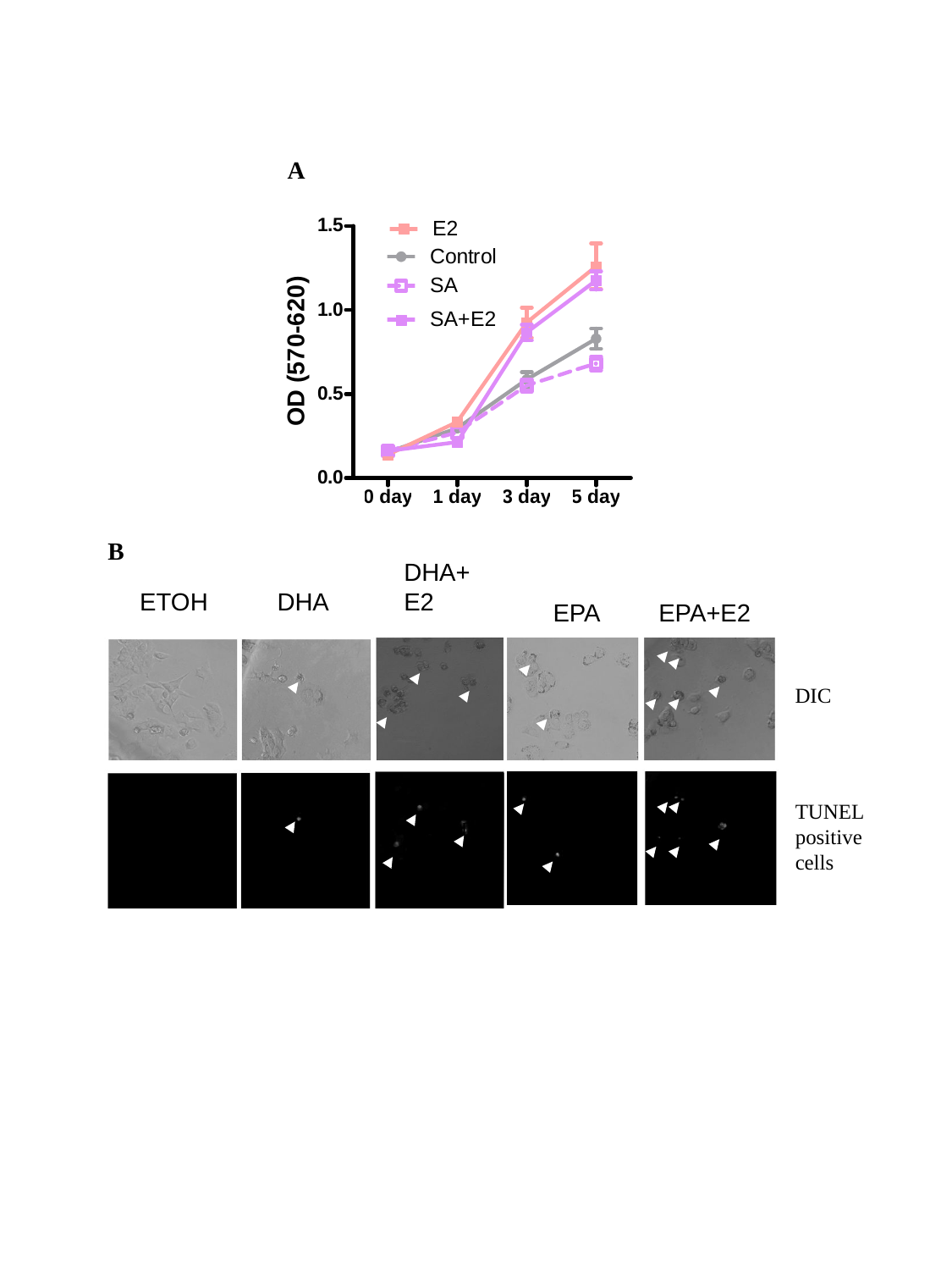

A
B
DHA+E2
ETOH
DHA
EPA
EPA+E2
DIC
DIC
TUNEL positive cells

Supplement: Figure S1 — A, Stearic acid (SA) does not promote the inhibitory effect of E2 in breast cancer cells. SA (90 µM) treated for indicated time points with or without E2. B, Images of MCF-7 cells after TUNEL Assay. MCF-7 cells treated as indicated for 72 hours, TUNEL assay was performed as described in Methods. Arrows indicated TUNEL positive cells. (PPT) [file pone.0052838.s001.ppt]

## Slide 1
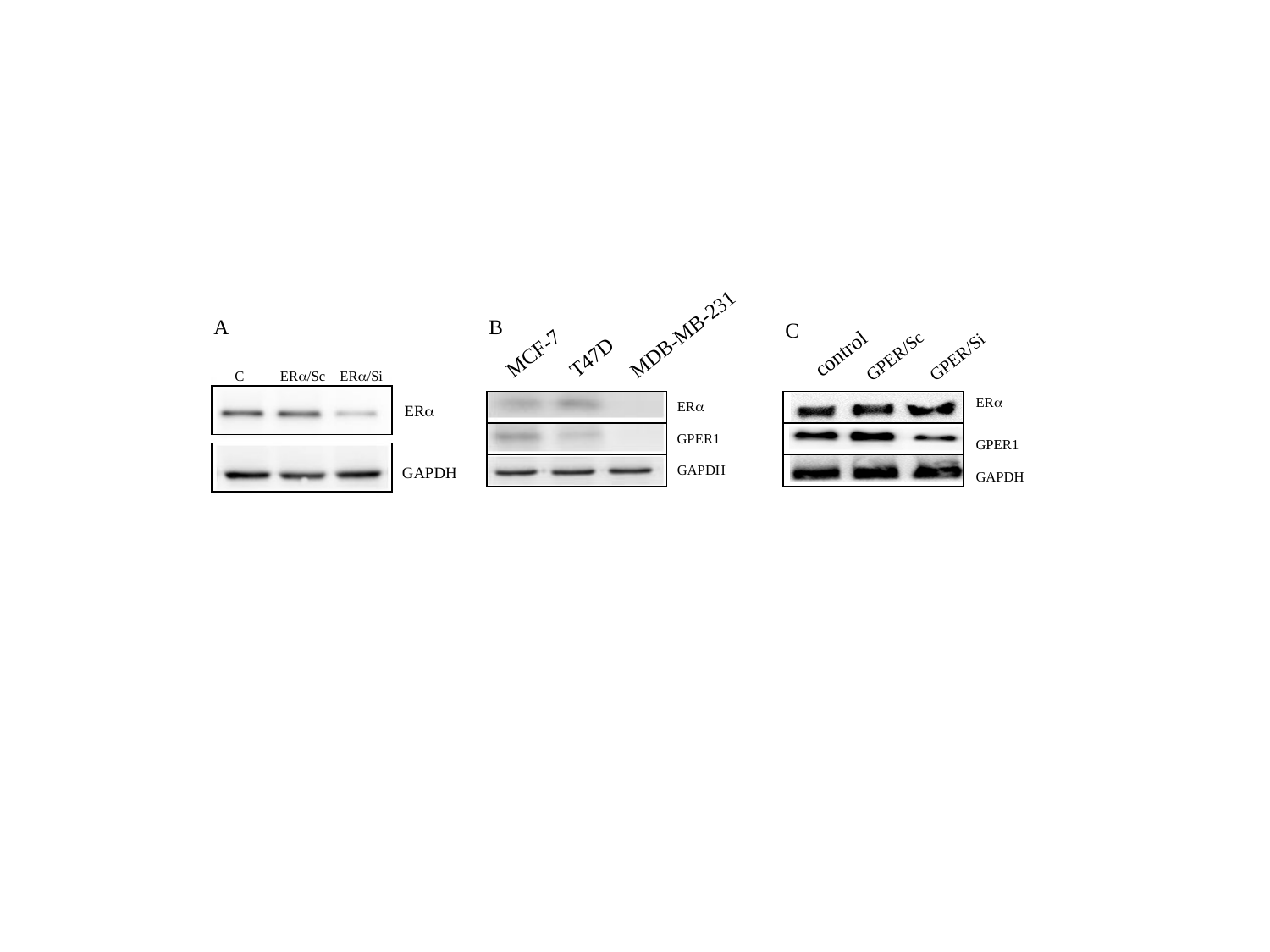

MDB-MB-231
A
 C ERα/Sc ERα/Si
ERα
GAPDH
B
C
control
MCF-7
T47D
GPER/Sc
GPER/Si
ERα
ERα
GPER1
GPER1
GAPDH
GAPDH

Supplement: Figure S2 — Protein expression in breast cancer cell. A, Western blot showed the expression of ERα in T47D cells after infected with lentivirus to deliver ERα or scramble shRNA. B, Western blot showed the expressions of ERα and GPER1 in indicated human breast cancer cell line. C, Western blots showed knockdown of GPER1 after transfection of GPER1 shRNA or control. (PPT) [file pone.0052838.s002.ppt]
