# Supplementary material for: N-3 Poly-Unsaturated Fatty Acids Shift Estrogen Signaling to Inhibit Human Breast Cancer Cell Growth
Source: PLoS One. 2012 Dec 28;7(12):e52838. doi: 10.1371/journal.pone.0052838 (PMC3532062; doi:10.1371/journal.pone.0052838)
Supplement: Figure S3 — MTT assay for BCa cell growth. A, MTT assay showed that 8-Bromoadenosine-3′,5′-cyclic monophosphorothioate, Rp-isomer (RP-cAMP, 10 uM), another PKA inhibitor, reversed the inhibitory effect of E2 on n-3 PUFA-treated MCF-7 cells (n = 3). B, 8-CPT-2me-cAMP (CT-cAMP), an agonist of cAMP-Epac signaling did not mimic the inhibitory effect of E2 on the n-3 PUFA-treated BCa cells (n = 3). C, n-3 PUFAs did not alter the ERα and GPER1 expression in n-3 PUFAs treated MCF-7 cells. MCF-7 cells were treated with DHA or EPA with/without E2 for 3 days. (PPT) [file pone.0052838.s003.ppt]

## Slide 1
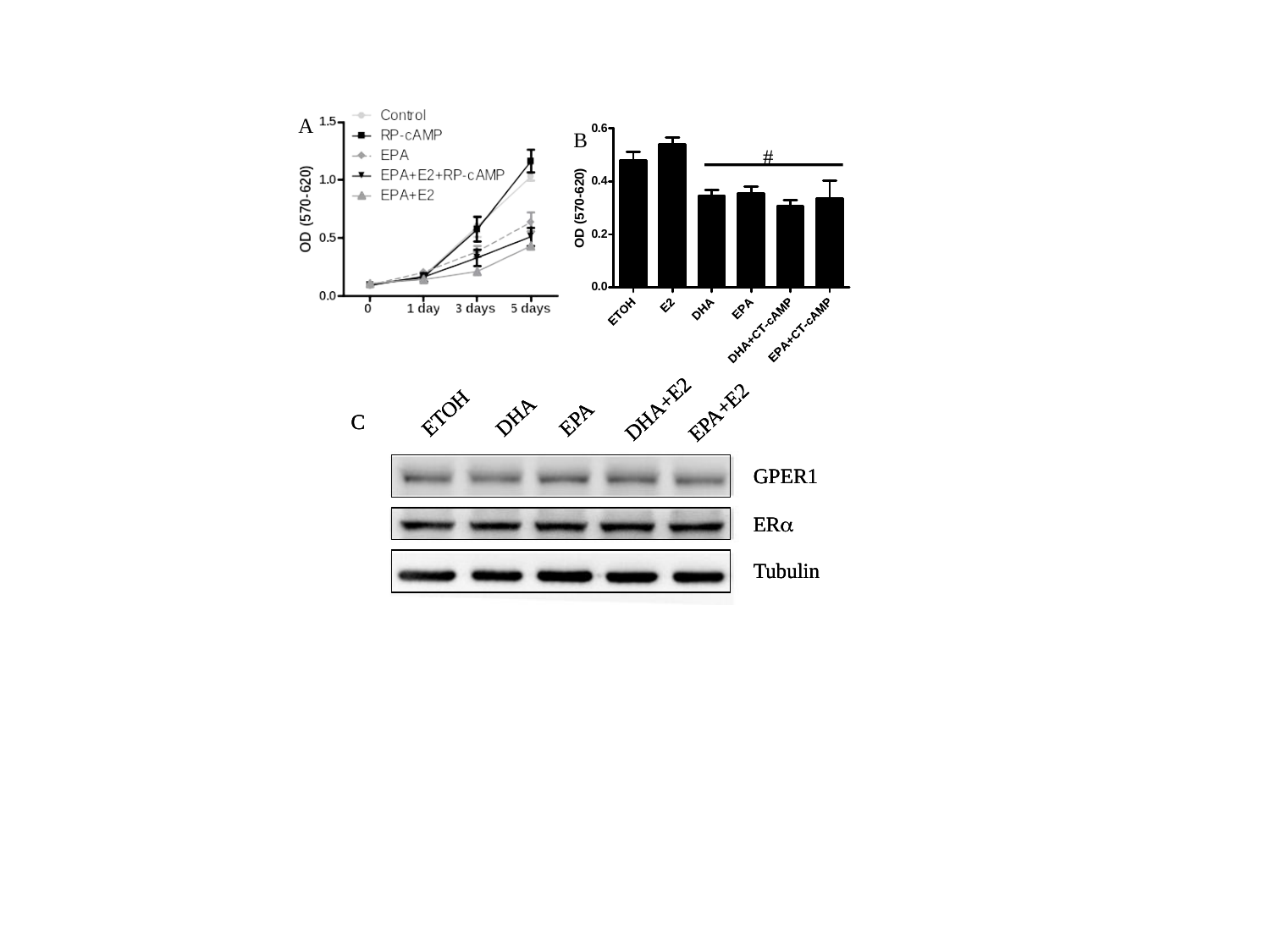

A
#
B
DHA+E2
EPA+E2
ETOH
DHA
EPA
GPER1
ERα
Tubulin
DHA+E2
EPA+E2
ETOH
DHA
EPA
GPER1
ERα
Tubulin
DHA+E2
EPA+E2
ETOH
DHA
EPA
GPER1
ERα
Tubulin
C
C
C
